# A topological data analysis method for revealing dynamic changes in psychotherapy microprocesses

**DOI:** 10.3389/fpsyg.2025.1711782

**Published:** 2026-01-22

**Authors:** Xiaochen Luo, Mengsen Zhang

**Affiliations:** 1 Department of Counseling Psychology, Santa Clara University, Santa Clara, CA, United States; 2 Department of Computational Mathematics, Science, and Engineering (CMSE), Michigan State University, East Lansing, MI, United States

**Keywords:** alliance ruptures, dynamical systems theory, interpersonal behaviors, interpersonal circumplex, temporal mapper, topological data analysis, psychotherapy microprocess, psychotherapy process

## Abstract

Understanding moment-to-moment therapeutic change is critical for advancing psychological interventions, yet existing tools rarely capture these dynamics. Dynamical systems theory offers a transtheoretical framework for modeling how therapeutic microprocesses shift and stabilize, but few methods can quantitatively link features such as stable states (“attractors”) and shifts (“transitions”) with empirical data, especially for high-dimensional systems when governing equations are unknown or unresolvable. We introduce Temporal Mapper, a topological data analysis (TDA) method that detects these features and represents their organization as attractor transition networks. As a proof-of-concept, we apply Temporal Mapper to psychotherapy microprocess data examining interpersonal behaviors and alliance ruptures. Our analyses revealed that therapist warmth stabilized dyadic interpersonal states within and between sessions, whereas confrontation ruptures stabilized dyadic interpersonal states within sessions but diversified and destabilized them across sessions. Beyond this example, Temporal Mapper offers a generalizable approach for uncovering fine-grained dynamic patterns, analyzing multimodal data of psychotherapy process, and identifying mechanisms of change at the system level to inform more effective interventions.

## Introduction

Psychotherapy is widely recognized as an effective way of facilitating psychological changes (Sakaluk et al., 2019). However, understanding how such transformation unfolds in the ebb and flow of each psychotherapy session remains a challenge, despite substantial efforts from empirical, theoretical, and clinical perspectives (Kazdin, 2009; Luo and Levendosky, 2025). Indeed, while psychotherapy researchers made consistent efforts to untangle the mechanisms of change at the between-session level, current research methods often struggle to model the complexity of psychotherapy at *fine-grained time scales*, while clinicians often rely on them to make clinical decisions in the moment. Unpacking the momentary, within-person processes of psychotherapy requires methodology that can model microprocesses that are time-intensive, multi-modal (e.g., Aafjes-van Doorn and Girard, 2025), non-linear (e.g., Hayes et al., 2007), and individualized (Moggia et al., 2023). Despite significant efforts, examining these within-person microprocesses remains surprisingly limited, especially among high-profile psychology and psychotherapy journals (Hopwood et al., 2025; Luo and Levendosky, 2025).

### The dynamical systems perspective to understand therapy microprocess

Dynamical systems theory (DST) is a mathematical framework for modeling multiple interacting processes and how the interaction gives rise to system-level patterns that cannot be reduced to independent components^1^ (Strogatz, 1994). While initially developed to understand how celestial bodies influence each other’s motions (Poincaré, 1967), in the last few decades, DST has been increasingly adopted in behavioral and biological sciences (Murray, 2007; Fuchs, 2013; Kelso, 1995; Tognoli et al., 2018), in particular to our interest, in modeling social interaction (e.g., Néda et al., 2000; Tognoli et al., 2020), including psychotherapy (Gelo and Salvatore, 2016; Hayes et al., 2007; Tschacher et al., 1998; Hoffart et al., 2025).

**Figure 1:**
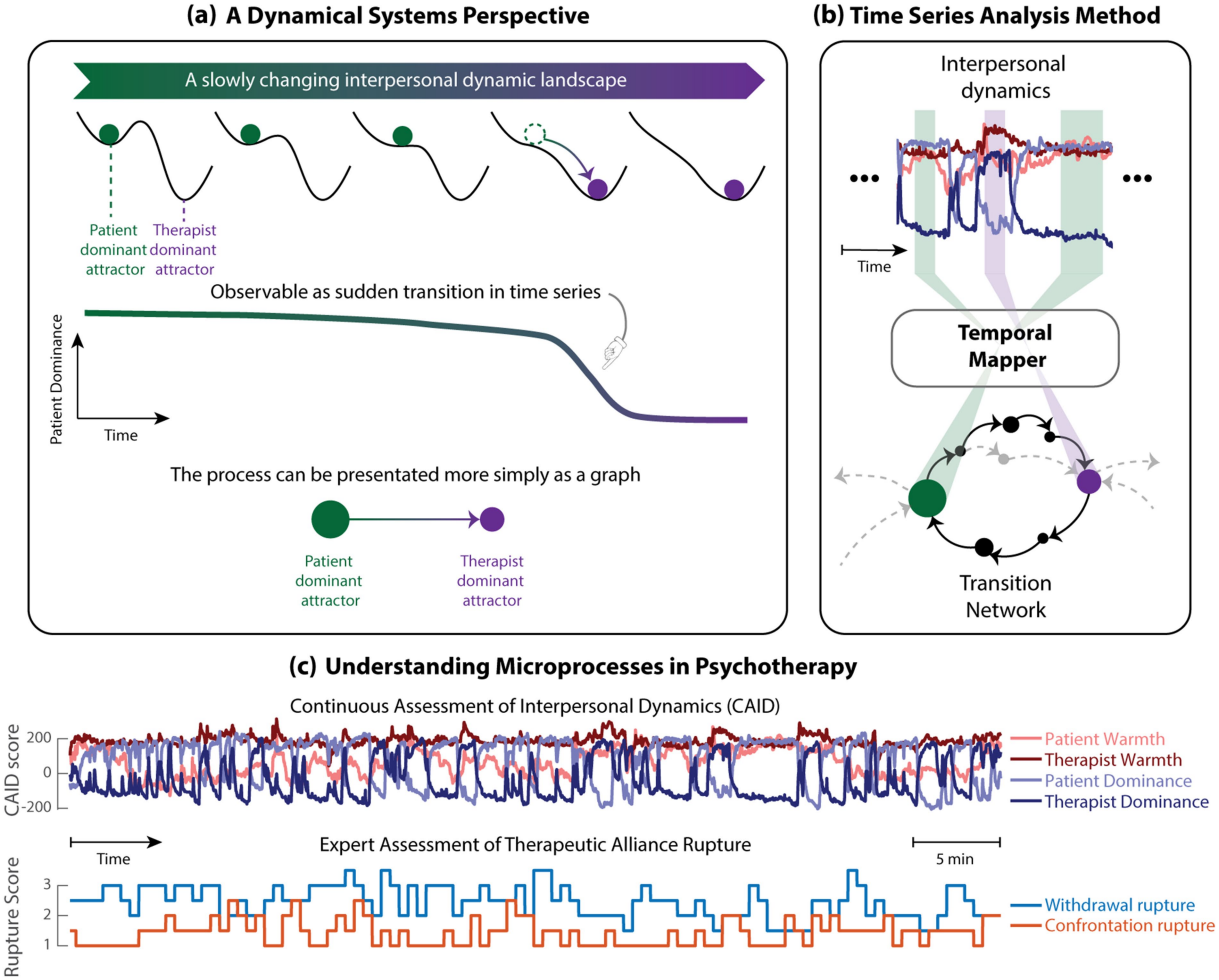
Introducing dynamical systems concepts into the analysis of interpersonal dynamics in psychotherapy. **(a)** A metaphorical illustration of the connection between dynamical systems concepts and state transitions observed in data (purely demonstrative purposes – not intended as an actual model of any interpersonal dynamics). In this cartoonish model, we consider the dynamics of the relative dominance between the patient and the therapist governed by a dynamical system, visualized as a landscape. The system has two stable states at the bottom of the valleys in the landscape: one when the patient is more dominant and the other when the therapist is more dominant. In dynamical systems language, such stable states are referred to as attractors. The pair can switch between those attractors when perturbed by noise or by slow changes in the shape of the landscape that destabilizes one of the attractors (4th landscape from the left). The landscape is not directly visualizable in higher dimensions with more than 1 or 2 variables, but the transitions can be seen in the time series of interpersonal dynamics (middle). Such transition between two attractors can be more simply represented as nodes connected by one directed edge (bottom). **(b)** Temporal mapper (Zhang et al., 2023) is a topological data analysis method employed in the present study to map 4-dimensional interpersonal states from the time series to nodes of a transition network. In this conceptual illustration, colored nodes denote attractors of specific interpersonal states; black nodes and paths denote paths of transitions between the two attractors; gray arrows and nodes reflect states and transitions outside of the time window. **(c)** (Top) Example time series of interpersonal dynamics in terms of warmth and dominance. (bottom) Example time series of moment-to-moment ratings of alliance rupture for the same session.

For a brief introduction, a dynamical system describes how the state of components evolves over time, depending on their current state and the law of interaction (a.k.a. equations of motion). Some states are especially interesting, which are called *attractors*, because the system tends to gravitate towards those states. For example, a state of rumination over a particular depressive thought may be considered a metaphoric counterpart of an attractor. In clinical literature (e.g., Scheffer et al., 2024), a dynamical system is often depicted pictorially as a landscape (Figure 1a, Top row), where the state of the system is represented by a ball rolling in the landscape. A valley represents an attractor, as the landscape will guide nearby states towards the local minimum. An attractor state is *stable* because if the ball is slightly perturbed away from the minimum, the landscape will guide it back. The extent to which the landscape can guide the system to return to a particular attractor is the *local stability*. However, the landscape can change as the context gradually changes. For example, if the ball represents moment-to-moment interpersonal states in therapy, the context could be the relationship between the two individuals. With the change of the relationship, the landscape can change so much that at one point a valley disappears (Figure 1a, top row, 4th landscape from left), meaning at this point the corresponding attractor has been *destabilized*. Since the state is no longer stable, the ball needs to find a new local minimum, thus a *phase transition* is triggered from the old attractor to the new attractor (Figure 1a, middle row). Such a transition is often observed in empirical time series data as a rapid change in state (Figure 1c, top row).

The moment-to-moment processes in psychotherapy are typically dynamic, non-linear, and under the influence of many interactive components, which are natural to model as dynamical systems.^2^ Existing dynamical systems models of psychotherapy processes typically take the form of differential equations (see Klocek et al., 2024 for a review), which are the mathematical counterpart of the metaphorical landscape (Figure 1a, top row).^3^ While some classical models are restricted to two dimensions (i.e., two interacting processes, e.g., Liebovitch et al., 2011), complex theories about the interaction between many processes have been translated into high-dimensional dynamical systems (e.g., Burger et al., 2020; Schiepek et al., 2016; Robinaugh, 2024). The development of high-dimensional dynamical systems models represents an important advancement in the theoretical study of mental disorders and their treatment, as they formalize traditional verbal or heuristic theories as differential equations, capturing multivariate interactions beyond mental tractability (Meehl, 1978; Robinaugh et al., 2021; Robinaugh, 2024). However, while high-dimensional systems could better capture the complexity of psychotherapy processes, they also come with challenges. First, once a dynamical system has more than 2–3 variables, it becomes difficult to derive what the system would *do* in all situations from the differential equations using traditional mathematical analysis (i.e., the equations become analytically intractable or insolvable). As traditional mathematical analysis becomes nonviable, understanding the behavior of high dimensional models generally rely on numeric simulations in a computer. It remains challenging, however, to understand the model’s full dynamic capabilities over the entire high-dimensional space. This is especially the case when the model has many attractors (i.e., extensively *multistable*) – imagine a high-dimensional landscape with a lot of valleys, where simulation often only reveals few of them, leaving most of the landscape uncharted (Zhang et al., 2019, 2022). Such complex dynamic landscapes have been observed and modelled in spontaneous social interactions (Zhang et al., 2018; Zhang et al., 2019, 2020; Tognoli et al., 2020), which is particularly relevant to our understanding of moment-to-moment interpersonal dynamics in psychotherapy. Second, the exact correspondence between features in the model dynamics and those in the empirical data also becomes difficult; thus, the comparison between mathematical models and empirical data are largely qualitative or rely on time-averaged measures, where one cannot systematically and quantitatively map each attractor in the model to each time point in the empirical data.

Here, we introduce a recently developed computational method, named Temporal Mapper (Zhang et al., 2023), which has been specifically designed to bridge the gap between high-dimensional dynamical systems models and empirical time series analysis. Temporal Mapper is a tool based on Topological Data Analysis (TDA), a growing field in applied mathematics aiming to quantify the “shape” of high-dimensional objects (see Ghrist, 2007; Carlsson, 2009, and Munch, 2017 for an introduction). While initially developed for geometric objects, TDA shows increasing utility in analyzing dynamical systems (Kalies et al., 2018; Saggar et al., 2018; Myers et al., 2019; Zhang et al., 2020; Tempelman and Khasawneh, 2020). Temporal Mapper was initially designed for characterizing high-dimensional dynamics in the brain as well as the dynamical systems models of the brain that often span hundreds to millions of dimensions. More specifically, Temporal Mapper detects attractors and transitions from time series and connects them into an *attractor transition network*, whose nodes map directly to attractors in the dynamical system and edges map to the transition between attractors (Figure 1b). While Temporal Mapper was developed to directly map features in the time series to those of dynamical systems models, it can be used as a stand-alone data analysis tool when *a priori* equations of motion are unknown, but the organization of attractors and transitions of the unknown system is nevertheless of interest. For example, quantitative features of the transition network can shine light on the local and global dynamics of the unknown systems. *Local dynamics* refers to how a state attracts other states that are very close to it, which can be studied in short time scales. With respect to psychotherapy, such local dynamics may be observed at the within-session, moment-to-moment scales (e.g., 1-second interval). *Global dynamics* refers to how different attractors relate to each other, e.g., how the system can transition from one attractor state to a very different one, possibly through many mediating attractors. Thus, global dynamics are usually observed on longer time scales. With respect to psychotherapy, changes in global dynamics may be observed at the between-session level. In this paper, we utilize an example to demonstrate how TDA can be used to understand complex psychotherapy processes at the within-session and between-session levels and for each individual and the whole sample.

### Example demonstration: the stabilizations and transitions of therapist’s and patient’s warmth and dominance and how they are related to alliance ruptures

Alliance ruptures refer to disruptions or challenges in the bond, or disagreement on goals and tasks between patient and therapist in psychotherapy (Eubanks et al., 2018). It typically includes two types: confrontation ruptures that often involve the two people “going against” each other, and withdrawal ruptures that often involve the two “going away” from each other. Alliance rupture has been recognized as a frequent therapeutic phenomenon that was influenced by the interactions between patient and therapist, and can be beneficial or detrimental to psychotherapy outcomes. Successful resolution of alliance ruptures enhances understanding of interpersonal needs of patients and contributes to enhanced therapy outcomes, whereas unresolved ruptures may lead to stagnant progress, long-lasting dissatisfaction, or even pre-mature termination (Eubanks et al., 2018). From a dynamical systems perspective, alliance ruptures can be seen as perturbations to the patient-therapist dyad, which may induce instability or phase transitions within the therapeutic relationship (Hoffart et al., 2025). Thus, metaphorically, alliance ruptures represent transitions in therapeutic alliance that may reflect shifts from a stable, collaborative state toward more maladaptive patterns (without repair of ruptures) or toward more adaptive relational dynamics (following successful repair of ruptures). In this sense, ruptures may serve as critical indicators of destabilization and reorganization in the therapeutic process that need attention (Gelo and Salvatore, 2016; Hayes et al., 2007; Salvatore and Tschacher, 2012).

To examine how the development and resolution of alliance ruptures may shift the therapeutic processes, it is essential to assess how the interpersonal behaviors of the therapist and patient manifest and interact with each other. Interpersonal behaviors have been conceptualized and assessed along the interpersonal circumplex, with dimensions of warmth/affiliation and dominance/control in the Contemporary Interpersonal Integrative Theory (Wright et al., 2023). The domain of warmth describes changes in interpersonal behaviors of getting more friendly or distant, whereas the domain of dominance describes changes in interpersonal behaviors of taking more charge or following more of other’s lead. In psychotherapy, the moment-to-moment assessment based on these dimensions (Continuous Assessment of Interpersonal Dynamics, CAID; Sadler et al., 2009) provides a fine-grained way to look at how each person’s interpersonal behavior changes in relation to the other person, and how the coordination of interpersonal behaviors of both patient and therapist lead to establishment, maintenance, disruptions, and repair of the therapeutic relationship. It is also worth noticing that although the concepts of confrontation ruptures and withdrawal ruptures were theorized based on different interpersonal motivations using the interpersonal circumplex, the empirical studies did not show significant associations at moment-to-moment level between specific types of observable interpersonal behaviors and specific type of alliance ruptures (confrontation or withdrawal), but rather identifying idiographic associations for each specific dyad and session (Luo et al., 2021, 2022). These results indicated that it may not be the change in single person’s specific behavior, but the coordination of interpersonal changes in both therapist and patient, that signal the changes in alliance ruptures and reflect the stability, destabilization, and phase transitions of therapy. To this date, such possibility of the associations between the system-level changes of interpersonal behaviors and alliance ruptures haves not been examined yet.

In this demonstration, we aim to show how Temporal Mapper can help reveal the complex relationships between alliance ruptures and interpersonal behaviors of warmth and dominance. We utilized a dataset containing intensive momentary assessments of patients’ and therapists’ interpersonal behaviors using interpersonal circumplex and within-session assessments of alliance ruptures. We illustrate how Temporal Mapper can be used to address three research aims. First, we demonstrated that it is possible to create a transition network for the dyadic interpersonal circumplex states, informed by four time series data of four dimensions of interpersonal behaviors (patient’s warmth, therapist’s warmth, patient’s dominance, and therapist’s dominance). The transition network can show the stability and shifts of the dyadic interpersonal system within each session. Second, we examined how the stability and shifts of the transition networks are related to individual interpersonal behaviors. Third, we examined how the stability and shifts of the transitional networks are related to alliance ruptures at within-session and between-session levels. Specifically, *within-session* changes refer to fluctuations occurring during a single therapy session, whereas *between-session* changes refer to session-to-session differences observed across the 16 sessions of treatment for each therapist–patient dyad.

## Methods of the example demonstration

### Transparency and openness

The dataset used in this study has been previously described in detail (Luo et al., 2022). All procedures were approved by the Michigan State University Institutional Review Board (Protocol #15–564) and conducted in accordance with ethical guidelines for human subjects research. The analytic code used to implement Temporal Mapper and reproduce the results reported here is publicly available at https://github.com/Multiscale-Complex-Systems-Lab/tmapper2.

### Sample and data characteristics

Briefly, outpatient psychotherapy video data were collected from a doctoral training community clinic focused on treating interpersonal problems and personality pathology. Videos of sixteen psychotherapy sessions featuring the highest alliance ruptures across the entire clinic were selected for this study from eight therapeutic dyads. We recruited all practicing therapists in the clinic, including eight therapists who were clinical psychology graduate students or pre-licensed staff clinicians trained to use an evidence-based, relational psychodynamic therapy approach, emphasizing the importance of the therapeutic relationship as a mechanism of therapeutic change. All therapists and their supervisors rated all their patients on frequency and intensity of alliance ruptures on a scale of 1 to 5, and the patient with the highest total score of alliance ruptures for each therapist was selected, resulting in eight therapeutic dyads of patient and therapist. The eight patients selected in the dataset were all self-referred to the clinic in a college town in a Midwest state of the U.S (62.5% male, 12.5% female, 25% identified as gender non-binary; *M*_age_ = 36.50, *SD* = 17.11; 87.5% White, 12.5% African American; 62.5% identified as heterosexual, 12.5% identified as gay man, 25% identified as queer). Two patients had attended college, while the other six patients had attended or graduated from high school. Five patients (62.5%) received a personality disorder as their primary diagnosis, while three patients (37.5%) received a primary diagnosis of major depressive disorder or dysthymia with significant relational difficulties.

Videos of all the therapy sessions between each selected patient and each therapist were then reviewed by an observer for alliance ruptures, and the two sessions with the highest alliance rupture ratings for each dyad were then selected, resulting in sixteen sessions in total in the sample.

### Measures

#### Interpersonal dominance and warmth

Details regarding the measures and training procedures have been reported previously (Luo et al., 2022). Briefly, the interpersonal behaviors of patient and therapist were assessed using the Continuous Assessment of Interpersonal Dynamics (CAID) on the dimensions of dominance and warmth relatively continuously by at least four trained coders (Sadler et al., 2009). The coder sees a computer monitor that displays the target video as well as a Cartesian plane depicting the interpersonal circumplex dimensions of dominance and warmth. A dot moves within the Cartesian plane in accordance with joystick movements, allowing coders to view the placement of their ratings as they watch videos. By using the joystick to move this dot, coders can indicate momentary shifts in interpersonal behaviors. The range of the scores is from −1,000 to 1,000 (extremely submissive/cold to extremely dominant/warm on the dominance/warmth dimension). The dominance and warmth scores are captured every half second by the computer program DARMA (Girard and Wright, 2018). Previous studies using this method reported Intra-Class Correlation Coefficients (ICCs) of 0.73–0.74 for dominance, and 0.43–0.44 ICCs for warmth (Dermody, et al., 2017). In the current study, the average ICCs across the sample for patient’s dominance and therapist’s dominance are 0.82 and 0.84, while the average ICCs for patient’s warmth and therapist’s warmth are 0.54 and 0.49.

#### Alliance ruptures

Alliance ruptures were measured at 30-s intervals using the Rupture-Resolution-Rating system (3RS; Eubanks et al., 2019) by four trained graduate coders with experiences of being a therapist. The 3RS system is an observer rating system designed to assess ruptures and repairs between therapist and patient. In the current study, it was modified to assess withdrawal ruptures (i.e., patients and therapists moving away from each other) and confrontation ruptures (i.e., patients and therapists moving against each other) at a 30-s interval instead of a 5-min interval as originally designed. Raters were asked to rate the intensity of confrontation rupture and withdrawal rupture separately for each 30-s interval on a scale ranging from 1 (no or little rupture) to 5 (extremely significant ruptures). Inter-rater reliability of ruptures was assessed by calculating the two-way mixed, average-measure, absolute agreement Intra-class Correlation Coefficients (ICC) for moment–moment coding (Hallgren, 2012). A suggestive ICC benchmark of 0.40 was used as a cut-off score for evaluating fair reliability of momentary coding (Cicchetti, 1994). For the current study, the average ICC across the sample was 0.60 for confrontation ruptures and 0.50 for withdrawal ruptures.

#### Topological data analysis (TDA)

##### Construction of the attractor transition networks

Temporal Mapper (Zhang et al., 2023) characterizes stable states (attractors) and transitions of nonlinear dynamical systems as a transition network (Figure 1b). The construction of the transition networks takes two steps. First, a spatiotemporal k-nearest neighbor (STKNN) graph is constructed from multi-variable time series data. The STKNN graph is a directed network, where each node corresponds to a time point in the time series. A directed edge connects two nodes if they are connected in time, and the direction of the edge reflects the direction of time. Two nodes are connected bidirectionally if they are close to each other in the state space (in this example, the 4-dimensional space consisting of the four interpersonal circumplex variables). The STKNN graph is a variation of the more familiar k-nearest neighbor (KNN) graph. A KNN graph can be constructed from any set of data points distributed in a high-dimensional space, where each point is connected to its top-*k* closest neighbors. Thus, k is the size of each node’s spatial neighborhood. A KNN thus represents the shape of the high-dimensional structure in the more tractable format of graphs, which provides the basis for further geometric or topological analysis (Van Der Maaten et al., 2009). However, KNN ignores any sequential information about the data; thus, the temporal information is lost when applying KNN to time series. A STKNN graph addresses the issue by explicitly incorporating the “arrow of time” in the neighborhood graph. In the second step, the STKNN graph is simplified by contracting nodes that are connected to each other within a distance *d* in the STKNN graph into a single node, producing a simplified graph. Here, the distance d is measured as the shortest path length in the STKNN graph, which can be interpreted as the minimal number of time steps to transition from one state to another; a larger d results in greater simplification and coarse-graining. This simplified graph is the attractor transition network, where each node maps to a set of time points. In the present study, we used neighborhood parameter k = 7 for STKNN graph construction and contraction parameter d = 3 to obtain the simplified graph for each psychotherapy session. The transition networks are chosen so that the construction is stable with small parameter changes, and further increasing k and d will reduce the complexity of the graph. After the transition networks are constructed, graph theoretical measures (such as the node size, the loop length) can be used to summarize the local and global topological properties of the graph.

##### Applying temporal mapper to interpersonal behavior data

Here, we apply Temporal Mapper to the analysis of dyadic interpersonal circumplex states, i.e., the time series of warmth and dominance for patient and therapist (e.g., Figure 1c, Top) in all 16 sessions. Temporal Mapper follows a general dynamical systems approach by characterizing system behavior as the relation between attractors and transitions, while remaining agnostic to the specific equations that generate them. Temporal Mapper detects the underlying attractor-transition structures based on the dynamic relations between states, rather than on the specific measurements or modality used to quantify the states. This feature makes Temporal Mapper a versatile tool applicable across disciplines and scales of measurement. More precisely, the ability of Temporal Mapper to extract attractors from the time series requires a separation of time scale (Zhang et al., 2023), meaning the transition between attractors or states (the transition is often referred to as an “escape”) is very sudden compared to any slow shifts within the attractors or states (referred to as a “dwell”). Such dwell-escape dynamics are apparent in the interpersonal dynamics time series (e.g., Figure 1c), enabling the stable interpersonal states to be portrayed as nodes in a network and the transition between them as edges (see Figure 1b for an intuitive illustration). These nodes are considered as attractors in a (hidden) interpersonal dynamic landscape (Figure 1a).

The size of nodes in the attractor transition network reflects the total dwell time in that attractor, which further reflects how stable a specific attractor is locally (how steep is the valley around a specific state). Such *local* stability is reflected by dynamics on a short time scale near a specific state. Thus, in the present study, local stability is compared across different attractor states within sessions. Meanwhile, the nodes, or attractors, are connected by paths of transitions (e.g., green attractor needs to move through three black attractors to reach the purple attractor in Figure 1b Bottom). The paths of transitions often form loops that connect an attractor back to itself. The *length of the loops* indicates how long it takes after the interpersonal dynamics have been perturbed away from an attractor to return to the same attractor. Thus, the loop lengths reflect a form of global stability over the dynamic landscape. In contrast to the local stability of a specific attractor, global stability concerns multiple, if not all, attractors and the difficulty of transitioning between them. Such global stability requires the analysis of more states over longer time scales. Thus, in the present study, global stability is assessed by cross-session comparisons.

As an overview, Table 1 provides explanations of how the terms in Temporal Mapper mapped onto traditional dynamical systems concepts and psychotherapy concepts in this example demonstration.

**Table 1:** The connections between Temporal Mapper, classical dynamical systems concepts, and psychotherapy process concepts in this example.

Classical dynamical systems terms (theoretical modeling method)	Temporal Mapper terms (analysis method)	Corresponding psychotherapy process (psychotherapy phenomenon)
Attractor	Nodes	Dyadic interpersonal circumplex states (4-dimension) that are returned to after small disturbance.
Ability to attract nearby states (attractor local stability)	Node size	How strong the dyadic interpersonal circumplex states are resistant to small disturbances.
Transition	Edge	The sudden change of a persistent dyadic interpersonal circumplex state into a qualitatively different state.
A series of transitions from one attractor back to itself	Loops	The process through which a dyadic interpersonal circumplex state recurs.
How many transitions it takes from one attractor to come back to itself (global stability)	Loop length	How easy it is for a dyadic interpersonal circumplex state to recur.

#### Analysis of connecting dynamical systems indices with interpersonal behaviors and alliance ruptures

Next, we performed further statistical analyses at within-session and between-session levels to understand the relationship between the transition network, specific interpersonal behaviors, and alliance ruptures. Nonparametric statistics (Spearman correlations and Wilcoxon rank sum test) are used in the study due to their compatibility with non-Gaussian distributions and their robustness against outliers. At the within-session level, we compute the Spearman correlation coefficient between the size of each node (i.e., local stability of each attractor) with interpersonal behavior variables (patient warmth, therapist warmth, patient dominance, therapist dominance) and alliance rupture variables (confrontation, withdrawal). The statistical analysis of the coefficients (*n* = 16) was performed at the group level using the Wilcoxon rank sum test, with the null hypothesis that the median of the distribution of the correlation coefficients is zero. A positive correlation means that the local stability of an interpersonal attractor is high when a specific variable has high values (e.g., high rupture).

At the between-session level, we first compute the session-average node size and loop length, as well as the average scores for interpersonal variables and rupture variables. High average node size and low average loop length indicate high local and global stability over all attractors of the dyadic interpersonal circumplex system. We then conducted Spearman correlations between session-average dynamical system indices (i.e., node size and loop length) and the average scores of interpersonal behavior variables and alliance rupture variables. A positive correlation means that the local or global stability over all interpersonal states is enhanced when a specific variable is high on average for a session (e.g., a session with persistent high warmth).

## Results of the example demonstration

### Visualization of the transition network for one session

**Figure 2:**
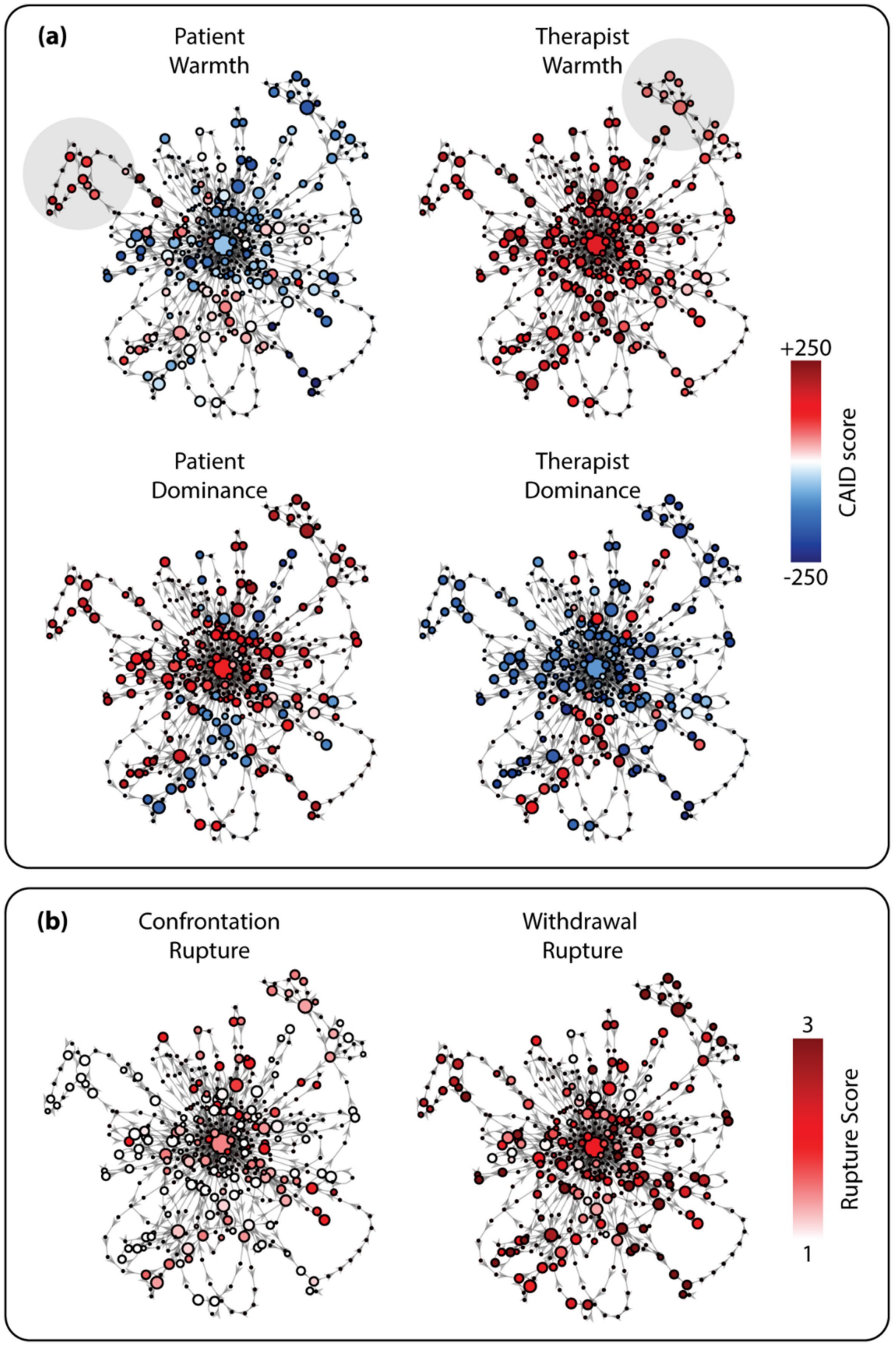
An example of an interpersonal state transition network for a single session constructed using Temporal Mapper. The graph was constructed from the CAID (continuous assessment of interpersonal dynamics) time series alone. In **(a)**, the nodes are colored by the average CAID scores associated with each node. The shaded areas highlight the example excursions where the interpersonal dynamics deviate from the central state (see text for details). In **(b)**, nodes of the same graph are colored by the average confrontation and withdrawal rupture of the associated time points. CAID scores range from −1,000 to 1,000 on both warmth and dominance dimensions. Positive (negative) dominance scores indicate dominance (submissiveness); positive (negative) warmth scores indicate warmth (coldness). Rupture scores range from 1 (no or little rupture) to 5 (extremely significant ruptures).

Using Temporal Mapper, a transition network of dyadic interpersonal circumplex states has been constructed for each session from interpersonal time series of patient warmth, therapist warmth, patient dominance, and therapist dominance. To show readers how the network can be interpreted, the transition network from one session is shown as an example (Figure 2), where the nodes represent attractive dyadic interpersonal circumplex states. While only visualizing one session, we conducted all subsequent analyses on the entire sample.

We colored each node using each specific interpersonal behavior variable (Figure 2a) to show how each node represents a particular combination of levels along each interpersonal circumplex dimension. Larger nodes indicate stronger interpersonal attractors, e.g., the large central node, where the therapist is warm and submissive, and the patient is dominant and a bit cold (Figure 2a). The central node is surrounded by various loops representing excursions from this central dyadic interpersonal circumplex state. For example, the top left loop reflects an increase in the patient warmth, and the top right loop a drop in the therapist warmth, which takes more time (a greater number of transitions) to reach and to recover from (shaded areas in Figure 2a).

To visualize the relationship between alliance rupture and the dyadic interpersonal circumplex states, we color the nodes of the same graph with the level of confrontation and withdrawal rupture, as shown in Figure 2b. It is noticeable that for this session, withdrawal ruptures and confrontation ruptures occur at different parts of the transition network. Nodes with high confrontation rupture (i.e., red nodes in Figure 2b left) are close to the central state, while in contrast, nodes associated with higher withdrawal rupture (i.e., red nodes in Figure 2b right) more often occupy the excursions on the outskirts in the peripheral areas. This indicates that in this session, the interpersonal circumplex states associated with higher confrontation ruptures are often easy to dwell in and to return to, compared to interpersonal circumplex states associated with higher withdrawal ruptures.

### The relationship between specific interpersonal behaviors and the transition network of dyadic interpersonal circumplex states

We examined how graph features – node size and loop length – of the transition networks are related to each of the four interpersonal variables (therapist warmth, therapist dominance, patient warmth, patient dominance). Recall that node size is a measure of *local stability* of specific interpersonal circumplex states (larger nodes → more local stability), and that loop length is a measure of global stability of the circumplex transition network (longer loops → less global stability). Due to its local nature, node sizes can be compared between states within a network (i.e., within a session) and between networks (i.e., between sessions). As a global measure of a transition network, loop lengths are used to compare across networks (i.e., between sessions).

**Figure 3:**
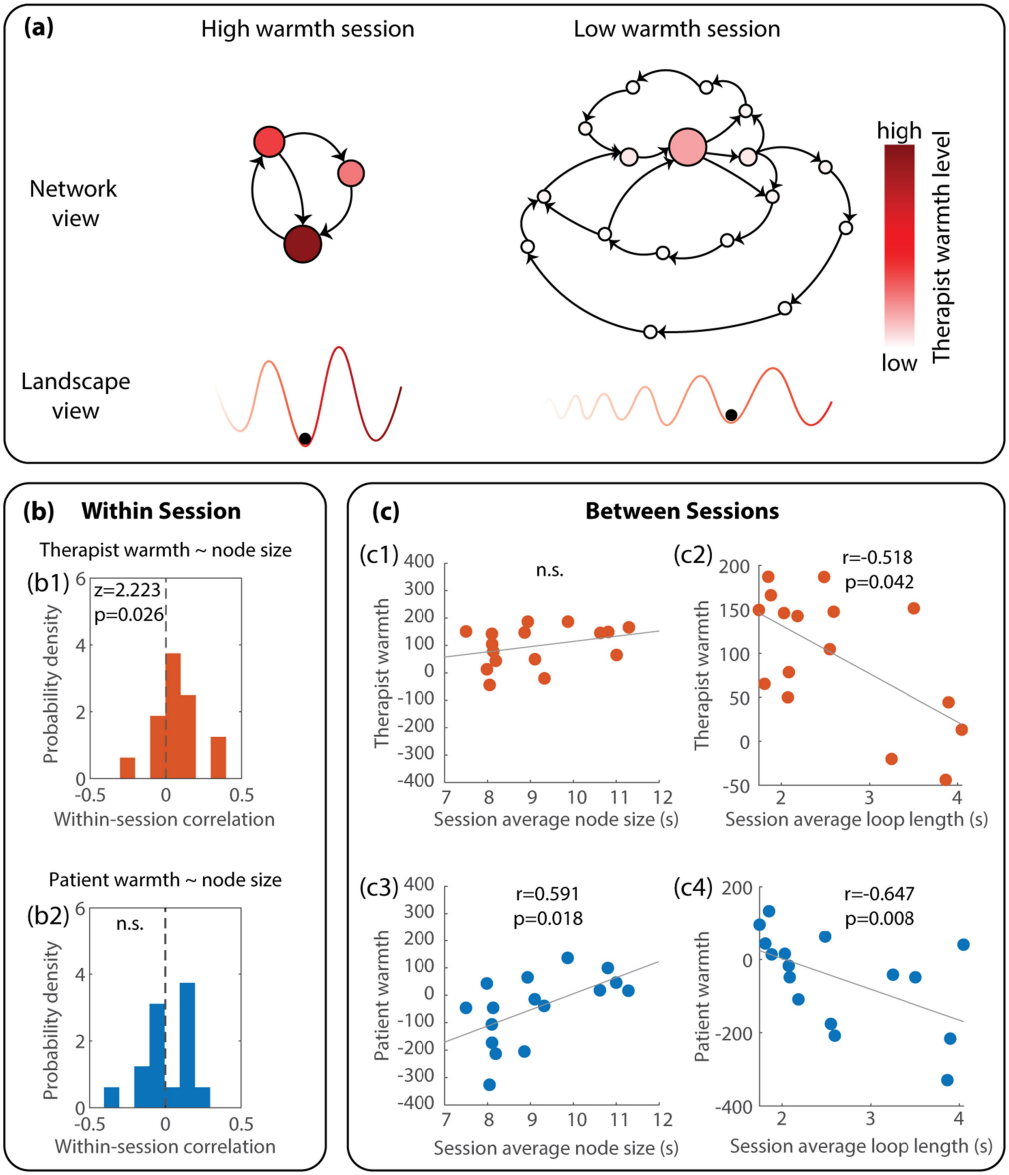
Therapist and patient warmth are associated with the stability of interpersonal dynamics at different scales. **(a)** Illustrates the main finding conceptually. Within sessions, higher levels of therapist warmth are found in larger nodes (darker red nodes within the network), suggesting a contribution to the local stability of interpersonal dynamics (deeper valleys in the landscape view with darker red). Between sessions, sessions with a higher average level of warmth from either the patient or the therapist are associated with shorter loops, suggesting an increase in global stability and a decrease in the diversity of interpersonal states (fewer valleys in the landscape view). **(b)** Shows the statistical results regarding the within-session correlation of the size of the nodes in the transition networks with the associated therapist warmth (b1) and patient warmth (b2). **(c)** Shows the statistical results regarding the between-session correlation of transition network properties (session-average node size, loop length) and session-average warmth of the therapist and the patient.

Within sessions, we found that only therapist warmth is associated with greater node size of the transition network (*z* = 2.22, *p* = 0.03, see Figure 3b1 for statistical results and Figure 3a for conceptual illustration), suggesting that high therapist warmth co-occurred with more stabilization of the interpersonal circumplex states. There is no significant correlation between node size and patient warmth (Figure 3b2, *z* = 0.42, *p* = 0.68), therapist dominance (not shown, *z* = 1.60, *p* = 0.11), or patient dominance (not shown, *z* = 0.10, *p* = 0.92). In other words, among all interpersonal circumplex variables, only therapist warmth is associated with local stability of the overall (four-dimensional) interpersonal circumplex states.

At the between-session level across 16 sessions, sessions with a greater average therapist warmth are associated with significantly shorter loops on average, suggesting an increase in global stability and a decrease in the diversity of dyadic interpersonal circumplex states (see Figure 3c2 for statistical results and Figure 3a network view for conceptual illustrations). Similarly, sessions with greater patient warmth are associated with both shorter loops and larger nodes on average (see Figures 3c3,c4 for statistical results and Figure 3a network view for conceptual illustration), indicating increased average local stability and global stability over all attractors. In other words, the interpersonal circumplex states were more persistent and easier to return to in sessions with warmer therapists/patients compared to sessions with colder therapists/patients. Translated into the landscape metaphor (Figure 3a, bottom row), it means that there are fewer, deeper valleys closely connected to each other (left) in warmer sessions (redder color).

In contrast to warmth, neither the dominance of the therapist nor the patient is significantly associated with any graph features (not shown; therapist dominance ~ average node size: *r* = 0.18, *p* = 0.51; patient dominance ~ average node size: *r* = −0.32, *p* = 0.23; therapist dominance ~ average loop length: *r* = 0.03, *p* = 0.91; patient dominance ~ average loop length: *r* = −0.15, *p* = 0.57).

### The relationship between alliance ruptures and the transition network of dyadic interpersonal circumplex states

**Figure 4:**
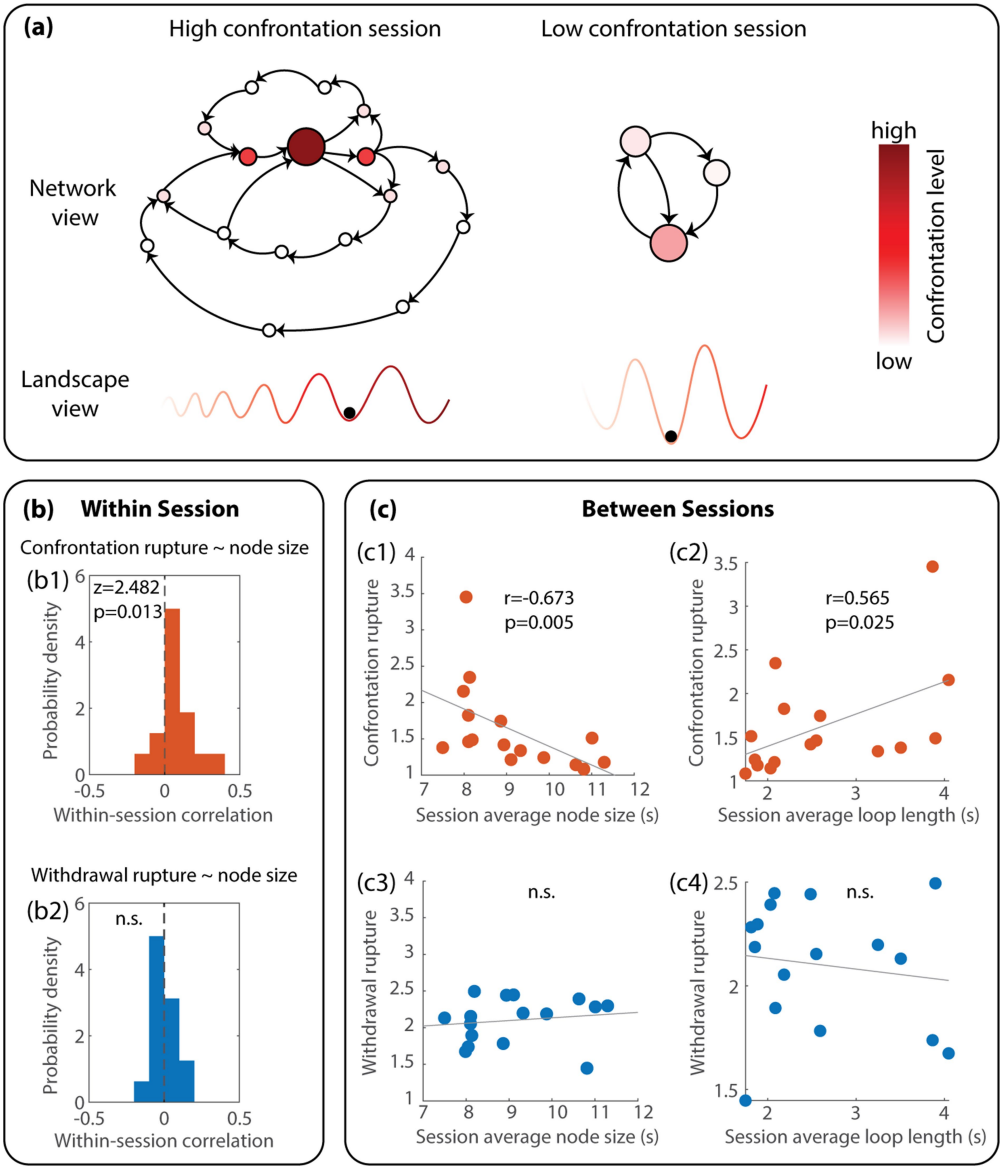
Confrontation and withdrawal rupture are associated with distinct interpersonal dynamics characterized by Temporal Mapper. **(a)** Illustrates the main findings conceptually regarding the features of interpersonal dynamics associated with confrontation ruptures. Namely, within sessions, high confrontation states are associated with larger nodes, i.e., they are stronger attractors (deeper valleys in darker red) in the landscape of interpersonal dynamics. Across sessions (between left and right networks or landscapes), high confrontation sessions had smaller nodes and longer loops on average, suggesting a globally flatter dynamic landscape with more possible attractors. No significant association was found between graph features and withdrawal ruptures; thus, it was not illustrated conceptually. **(b)** Shows the corresponding statistical results of within-session correlations using the Wilcoxon signed rank test for both confrontation and withdrawal rupture. **(c)** Shows the statistical results of between-session correlations between graph features (session average node size and loop length) and rupture levels using Spearman correlation for both confrontation and withdrawal rupture. Note that the scatter plots are shown in original scales only to give the reader a sense of actual values – the actual statistics are based on rank. What may appear to be outliers in the original scales are not actually outliers in rank (see Supplementary Figure S1) for rank-based statistics.

At the within-session level, high confrontation ruptures tend to occur in larger nodes in the transition network (*z* = 2.48, *p* = 0.013; see Figure 4b1 for statistical results and Figure 4a network view for conceptual illustration), meaning locally more attractive interpersonal states co-occur with higher confrontations within each session. This indicated that the dyadic interpersonal circumplex states tend to stabilize when higher confrontation ruptures occur. Translated into the landscape metaphor (Figure 4a, bottom row), this means that within the same landscape, valleys associated with higher confrontation ruptures (darker red) are deeper. Across sessions, however, sessions with higher average confrontation ruptures are associated with a smaller average node size (*r* = −0.67, *p* = 0.005) and a greater average loop length (*r* = 0.57, *p* = 0.03) (see Figures 4c1,c2 for statistical results and Figure 4a network view for conceptual illustration). This means that at the between-session level, sessions with higher confrontation showed reduced average, global stability and increased diversification of dyadic interpersonal circumplex states. In the landscape view, this means that higher confrontation sessions (Figure 4a, bottom left) are associated with more, but on average shallower, valleys. In summary, confrontation ruptures were associated with greater local, short-term stability of specific dyadic interpersonal circumplex states at the within-session level, but also associated with decreased global, long-term stability and diversified interpersonal states at the between-session level.

We conducted post-hoc analyses to explore the associations between therapist/patient warmth and confrontation ruptures at within- and between-session levels, given that they were both significantly correlated with node size and loop lengths. We found that at the within-session level, there is no significant correlation between therapist warmth and confrontation rupture (*z* = −1.29, *p* = 0.20), suggesting that each of them was independently associated with node sizes at the within-session level. At the between-session level, sessions with greater confrontations were significantly associated with lower patient warmth (Spearman’s rho = −0.50, *p* = 0.035) and marginally lower therapist warmth (*Spearman’s rho* = −0.497, *p* = 0.052). This suggests that warmth and confrontation may work together in balancing overall stability of the landscape and diversity of interpersonal dynamics: in sessions with higher confrontations and lower warmth of either patient or therapist, there tends to be more diverse and less stable dyadic interpersonal circumplex states, whereas, in sessions with higher warmth of patient or therapist and less confrontation ruptures, the dyadic interpersonal circumplex states are more stable and less diverse.

In contrast, we found that withdrawal rupture greatly differs from confrontation ruptures: withdrawal ruptures are not significantly related to any features of the interpersonal circumplex transition network, either within sessions (Figure 4b2, *z* = 0.26, *p* = 0.80) or between sessions (Figures 4c3,c4; *r* = 0.40, *p* = 0.13 for correlation with node size, *r* = −0.13, *p* = 0.63 for correlation with loop length). This means that withdrawal is not associated with attractors nor transitions in the dynamics of dyadic interpersonal circumplex states.

## Discussion of the example demonstration

In the present example, we employed a TDA method, namely Temporal Mapper, to characterize the dyadic interpersonal circumplex states between patient and therapist during and across psychotherapy sessions. We computationally constructed the transition network from empirical data of interpersonal behaviors of patients and therapists. Analyses of network features reveal distinct roles of the therapist’s warmth and confrontation ruptures in stabilizing and destabilizing the dyadic interpersonal circumplex states at different scales.

### The relationship between dyadic interpersonal circumplex states and each specific interpersonal behavior variable

At the within-session level, higher therapist warmth, but not other interpersonal circumplex variables, is related to more stable dyadic interpersonal circumplex states. This suggests that therapist warmth is a moment-to-moment thermometer of the stability of the dyadic interpersonal states. At the between-session level, both therapist’s and patient’s warmth are positively related to the global stability of the dyadic interpersonal circumplex states. Thus, higher average warmth of patient or therapist seemed to also serve as global markers for sessions with increased global stability and decreased diversification of dyadic interpersonal states.

While further causal inferences are beyond the scope of the present paper, the results were consistent with the theory of the possible role of therapist warmth in serving an immediate holding and stabilizing environment for patient in the moment and across sessions in therapy (Ackerman and Hilsenroth, 2003). These findings also pointed to the role of warmth between patient and therapist – sessions with higher pre-treatment levels of warmth are often shown to be related to better treatment outcomes and fewer dropouts (McFarquhar et al., 2018), which may be achieved through stabilizations of adaptive patterns and co-regulations of dysregulated affects/behaviors. On the other hand, future research may explore when warmth may not be as beneficial through over-stabilizing the system, especially when destabilization of maladaptive patterns such as interpersonal impasses is needed.

In contrast to the role of warmth, the dominance of either the therapist or the patient does not play any role in the stability of dyadic interpersonal states. This finding is somewhat consistent with the unclear, mixed role of dominance played in psychotherapy: unlike the clear, beneficial role of warmth in stabilizing therapy, mixed results have been found for the role of dominance in strengthening or dissolving the therapeutic relational work (McFarquhar et al., 2018). Future studies should further explore the role of dominance in the therapeutic dynamical systems.

### The relationship between alliance ruptures and dyadic interpersonal circumplex states

We found that confrontation rupture related differently with local and global stability of the dyadic interpersonal circumplex states: within each session, stronger attractors were typically associated with higher confrontations; In contrast, at the between-session level, sessions with higher confrontation ruptures were associated with more diverse, less stable dyadic interpersonal circumplex states and longer transitions between these states, compared to sessions with lower confrontation ruptures. It is possible that in the moment, a confrontation may catalyze itself from the reciprocating negative affect of both parties and can attract nearby states to evolve towards it, a phenomenon that has been documented through the dynamical systems modeling of couple dynamics (Gottman et al., 2002) and affective interactions in psychotherapy (Liebovitch et al., 2011). In other words, the dyad tended to dwell in the states associated with higher confrontation ruptures within the session. However, in the long run, confrontation ruptures may also push the therapeutic dyad to explore more diverse interpersonal states that other dyads with fewer confrontation ruptures have rarely explored. The finding at the global scale supported and extended the literature in suggesting the double-edged role that confrontation ruptures may play in the stability of psychotherapy (Eubanks et al., 2018). On the one hand, confrontation ruptures brought variability to the dyadic system, which may be a potential benefit of creating more chances to express unmet needs, letting the patient and the therapist explore a more diverse range of interpersonal states when progress is stagnant. On the other hand, such an increase in the exploration of interpersonal states can also increase the chances of entering a specific attractor – the pre-mature termination of the therapeutic relationship, which can be detrimental to the therapeutic progress.

Given that both warmth and confrontation ruptures were related to node size and loop length in the transitional network, we explored how the two may co-shape the dyadic interpersonal circumplex states at within- and between-session levels. Within sessions, confrontation and therapist warmth independently related to increased stability of the interpersonal circumplex states—this means that patient and therapist frequently returned to familiar interpersonal circumplex states during moments of intense confrontation or expressions of warmth in an intense session. Perhaps they followed more inter-locked patterns (for either the impasse during conflicts or the stabilization during warmth expression). However, at the between-session level, warmth and confrontation have the opposite associations with increased or decreased global stability of the interpersonal circumplex states. These results suggest paying attention to the possible balance between using warmth to stabilize and making use of the confrontation to diversify the therapeutic interactions: it is possible that reciprocal warmth can stabilize the interpersonal dynamics when the relationship is not strong but may prevent the pair from exploring new interpersonal states during a therapeutic impasse, whereas confrontation can be beneficial for exploration of new dyadic interpersonal states at the cost of reduced stability and the possibility of transiting to other attractor states such as pre-mature termination. Thus, a key question for future study is how the increased or decreased global stability of interpersonal circumplex states may be reached for optimal therapeutic change.

In contrast, withdrawal rupture is not associated with any dynamic features represented in the transition network. In a sense, withdrawal is manifested as a form of *stalling*, as it does not bring new information into the interpersonal circumplex states, nor does it maintain the stability of the states. This diffused nature of withdrawal ruptures is consistent with the literature in suggesting the different roles of confrontation and withdrawal rupture—while confrontation rupture demands more interpersonal regulation, withdrawal ruptures activate more intra-personal regulation for the patient but little for the dyadic system (Tchizick et al., 2024), and are less noticeable by therapists (Kline et al., 2018).

### Limitation of the example demonstration

Our example demonstration has some limitations. First, our dataset contains only 16 sessions of interpersonal dynamics; this is because the use of dynamical system models typically does not rely on a large sample size but more on intensive assessment intervals for each case. However, the results should be further validated in a larger dataset. Second, the assessment of interpersonal dynamics and alliance ruptures is labor-intensive, and the reliability of therapist warmth remains relatively low (although compatible with the literature), partly due to the smaller variability in therapist warmth. However, it is worth pointing out here that the stability or persistence of a (four-dimensional) interpersonal circumplex state or attractor cannot be determined solely by small variability in therapist warmth. This is because for the interpersonal circumplex state to remain in an attractor, all four variables must persist at the same level. In fact, if one variable is constant while not affecting how the other three vary over time, the one constant variable is effectively decoupled from the dynamical system – one would obtain the same transition network if the constant variable were removed. Thus, the association between warmth and the enhanced stability of circumplex states must be mediated by the stabilizing influence of therpaist warmth on the dynamics of other interpersonal circumplex variables.

Third, we could not explore the longitudinal changes of the transition networks and how this may relate to treatment outcomes, nor did we explore how the repair of ruptures may be related to the transition network. Fourth, to reduce false positive results, we only picked several key metrics instead of other common graph theoretical measures (e.g., betweenness, hubness, small-worldness, etc.) to characterize the features of the transition networks. Lastly, this is a case demonstration; therefore, we did exploratory analysis instead of having a prior hypothesis.

## General discussion

### Advantages and disadvantages of Temporal Mapper over existing methods

The main advantage of Temporal Mapper is that it provides a means to capture attractors, transitions, and their global organization in very high-dimensional dynamical systems where the equations of motion are unknown. This is important for modeling psychotherapy processes and other real-world social interactions because their complexity cannot be fully captured with a handful of state variables and equations. Even if one has discovered this full set of equations, the models themselves are not fully understandable in terms of their organization of attractors and transitions without developing new computational tools—indeed, Temporal Mapper was first developed to understand complex high-dimensional neurodynamic models (Zhang et al., 2022, 2023). The feature of directly capturing the dynamic organization of the system without knowing the equations opens up more avenues to explore the dynamical systems modeling of psychotherapy processes by combining different variables and modalities in relation to specific clinical outcomes of interest. Moreover, in contrast to traditional dynamical systems modeling, which requires substantial training in relevant mathematics and computation, Temporal Mapper is relatively much easier to use, lowering the barrier for experimental and clinical researchers to utilize dynamical systems tools and concepts. Finally, Temporal Mapper shares the advantage of traditional dynamical systems approaches in capturing nonlinear, synergistic aspects of multivariable interactions that are not reducible to independent, linear relations between pairs of variables.

In addition to data-driven modeling demonstrated in the present work, Temporal Mapper can also serve as a bridge between equation-driven and data-driven modeling of interpersonal dynamics in psychotherapy. When the differential equations describing interpersonal interaction are available, one can use simulations and other equation-based stability analyses to construct an equation-based attractor transition network, which can then be compared to the data-driven transition networks illustrated in the present work [see technical details in Zhang et al. (2023) and Chowdhury and Mémoli (2019)].

Nonlinear modeling methods are always a double-edged sword—here, Temporal Mapper is no exception. On the one hand, users of such nonlinear methods aim to derive complex conclusions to capture real-world complex phenomena, while, at the same time, scientists’ minds instinctively search for simple explanations. Building on the basic framework presented in this paper, further analysis using Temporal Mapper can reveal more subtle and complex relationships, as discussed below, which is both an advantage and a disadvantage, especially for those seeking simple explanations. We do not anticipate that from Temporal Mapper one would eventually derive a single, universal explanation of all psychotherapeutic mechanisms. Rather, Temporal Mapper should be treated as a sort of “microscope”—it could show many new things in detail that may not have been conceptualized in macroscopic, time-averaged analysis of psychotherapy, but more work needs to be done to build on such newly gained perspectives.

### Potential future applications for psychotherapy research and clinical practice

First, Temporal Mapper can be used to explore temporal precedence and reveal the pathways leading to, and moving from, desirable (e.g., sudden gains, innovative moments) and undesirable states (impasses, increased suicidal risks) in psychotherapy. Thus, this exploration of the transition in psychotherapy may help clinicians elucidate ways to disrupt undesirable interpersonal dynamics and to enter desirable ways of interaction.

Second, Temporal Mapper can be used to capture *decisive* states—watersheds leading to two or more distinct attractors, thereby leading the interpersonal system to diverge into significantly different outcomes. Identifying these decisive states is even more important than the individual paths leading to undesirable attractors because, in such a state, the smallest intervention can greatly impact the outcome.

Third, Temporal Mapper can illuminate transitions and shifts not only within sessions but across multiple sessions. Using such a multi-session network, one can examine how system-level changes may lead to effectiveness at the whole treatment level.

It is important to note that Temporal Mapper can be applied not only to the interpersonal variables of dominance and warmth, but also broadly to understanding oscillations and coordination of other variables that are important in psychotherapy microprocesses (Luo and Levendosky, 2025). For example, researchers of affective processes can examine how the affective arousal and valence of both patient and therapist oscillate together, and how the changes in the affective system may be associated with behavioral changes. Another example of examining cognitive microprocesses may be using Temporal Mapper to delineate the change trajectories of the dyadic systems in patient’s and therapist’s mentalizing processes and examine how the changes of the systems may relate to therapy outcomes.

Temporal Mapper is not a *specific* dynamical systems model that attempts to provide a mathematical model (e.g., differential equations) and their conceptual/theoretical framework to interpret the process of psychotherapy (an example of this, see perceptual control theory Higginson et al., 2011). Rather, Temporal Mapper follows a general dynamical system approach, being agnostic about the specific equations and material interpretations underlying the relations between attractors and transitions. Thus, it can be a transtheoretical tool to examine the coordinated changes of multiple important processes that matter for specific theoretical orientations (a list of microprocesses being examined for specific orientations, see Luo and Levendosky, 2025). For example, researchers of motivational interviewing may be interested in the transition network of change talk and sustain talk in both patients and therapists and how the changes in the transition network may lead to changes in alliance and therapy outcomes. Importantly, Temporal Mapper’s capacity to capture attractors and transitions in non-linear systems makes it a promising tool to model multi-modal data that are increasingly popular in data collection of psychotherapy processes. As researchers continued to collect multimodal data of observational and physiological psychotherapy microprocess (e.g., linguistic content, affective process, behavioral intervention, HRV, etc), Temporal Mapper would provide a strong tool to analyze the non-linear trajectories of these psychotherapy processes at a systemic level.

### Theoretical implications based on dynamical systems theory

**Figure 5:**
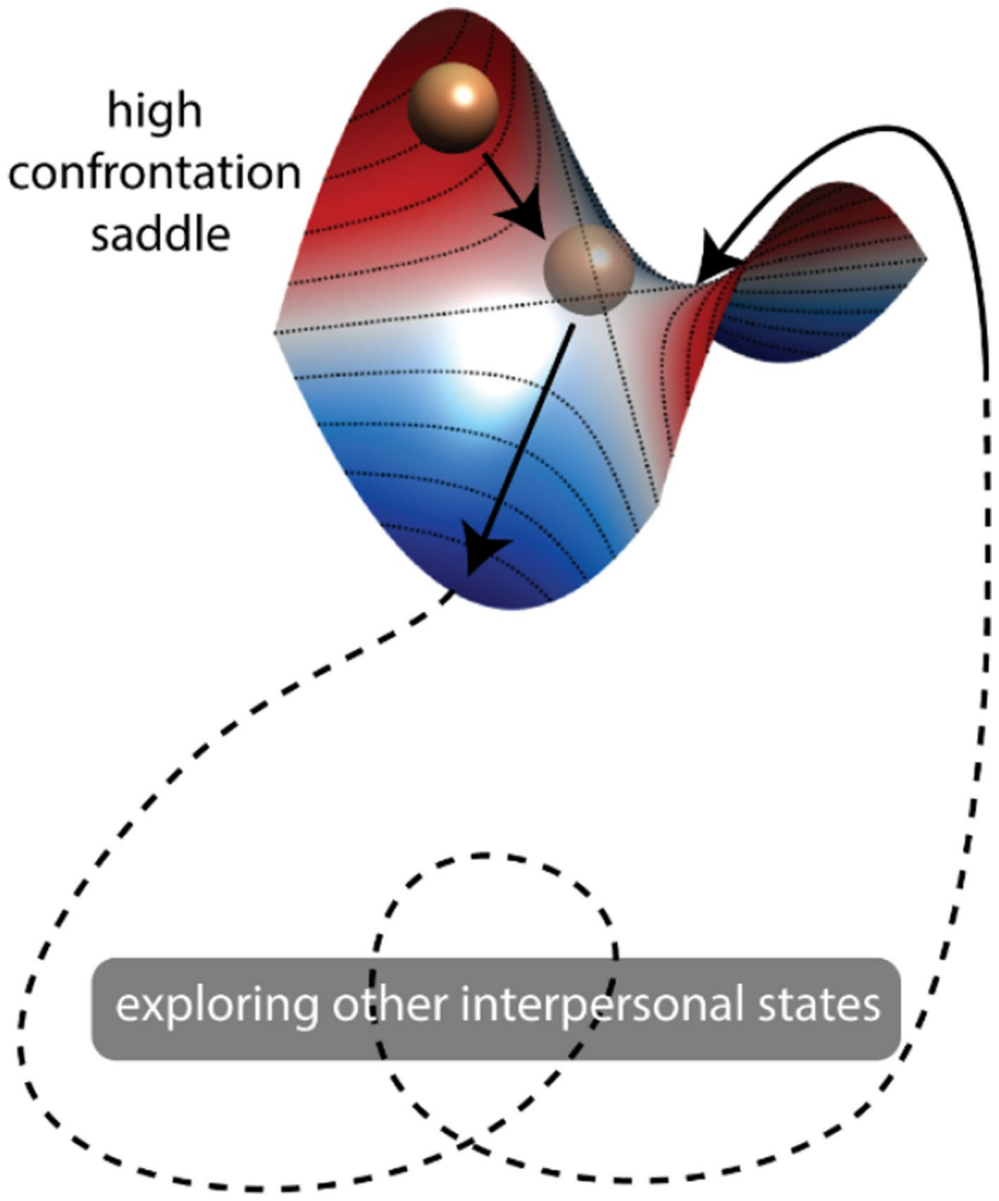
Intuitive illustration of saddle-like dynamics locally and globally. A hypothetical saddle is shown representing a high-confrontation state. The elevation in the local landscape of the saddle was indicated by colors (red = high, blue = low). The very center of each saddle is an equilibrium point, i.e., a system can stay there forever if unperturbed. The red ridge indicates the stable direction of the equilibrium – if the state of the system (ball) starts on this red ridge, it will be *attracted* to the equilibrium (rolling down towards it). The blue valley indicates the unstable direction – if the system is slightly perturbed, it will move away from the equilibrium. Thus, a saddle is both attractive and repelling. From a more global perspective, the high confrontation saddle can attract the dynamics towards it but then spontaneously push the system away along the unstable (blue) direction, leading to the exploration of different interpersonal states (dash lines indicate unshown part of the landscape where there may be more saddles).

From the dynamical systems theory perspective, the complex role that confrontation ruptures played in the system may be conceptualized as *saddles* (Figure 5). A saddle is a state in a dynamical system that attracts the system towards it from some directions (a.k.a. stable directions) while repelling the system away from it in other directions (a.k.a. unstable directions). Our findings suggest that confrontational states have a stronger attractive direction locally (Figure 5, high confrontation saddle, red ridge), while at the same time, they push the system away to explore other states (Figure 5, dashed lines). Such saddle-driven dynamics are rarely examined in psychotherapy literature [see recent reviews by Hayes and Andrews (2020), and Klocek et al. (2024)], while continue to gain attention in other domains such as neuroscience (Kelso, 2012; Tognoli and Kelso, 2014; Hancock et al., 2025). In the present study, we provide some initial evidence pointing to the possibility that *confrontation rupture creates saddles in interpersonal dynamics, leading to enriched global dynamics*. Further theoretical studies are required to investigate what features of the transition networks constructed using Temporal Mapper best differentiate between *attractors vs. attractive saddles.*

## Conclusion

To conclude, we have introduced a new TDA method, Temporal Mapper, to examine the interpersonal dynamics during psychotherapy and how it may relate to the rupture of the therapeutic alliance. Our initial explorations suggest that confrontation and warmth are opposing forces that modulate the global stability and diversity of interpersonal dynamics: confrontation ruptures destabilize the system and promote exploration of more diverse interpersonal states that may lead to drop-outs or growth, while warmth, especially therapist warmth, stabilized the interpersonal system at fewer interpersonal states that may facilitate regulation or over-stability. We demonstrated the potential of this novel quantitative tool to delineate system-level transitions and their impacts on therapeutic relationships and outcomes. In the long run, this work provides the mathematical and computational basis for comparing dynamics across treatment paradigms and across modalities at intrapersonal, interpersonal, and neurophysiological levels.

## Data Availability

The data analyzed in this study is subject to the following licenses/restrictions: Temporal Mapper tools used in the paper are publicly available at https://github.com/Multiscale-Complex-Systems-Lab/tmapper2. Please direct questions regarding software usage to MZ. Requests to access these datasets should be directed to XL, xluo@scu.edu; MZ, mengsen@msu.edu.

## Footnotes

1 Here we are only concerned with nonlinear dynamical systems. For simplicity, in this manuscript, we will only refer to them as dynamical systems.

2 For simplicity, we refer to nonlinear dynamical systems as dynamical systems throughout the present manuscript. Due to the limit of space, we will not further discussion the technicality of nonlinearity.

3 Not all dynamical systems can be represented as a high-dimensional landscape. The landscape metaphor is here used to provide non-expert readers intuition regarding the relationship between differential equations, attractors and transitions.
