# Supplementary figures and images for: A topological data analysis method for revealing dynamic changes in psychotherapy microprocesses
Source: Front Psychol. 2026 Jan 22;16:1711782. doi: 10.3389/fpsyg.2025.1711782 (PMC12872820; doi:10.3389/fpsyg.2025.1711782)

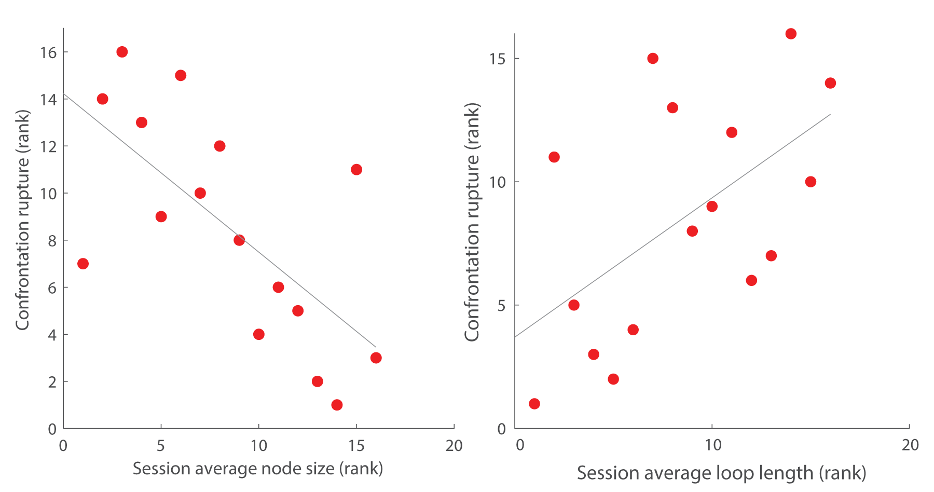

Supplement: Supplementary file 1 [file Image_1.png]
